# ﻿Pholcid spiders of the genus *Pholcus* Walckenaer, 1805 (Araneae, Pholcidae) from eastern Sichuan, China, with descriptions of two new species

**DOI:** 10.3897/zookeys.1264.175373

**Published:** 2025-12-11

**Authors:** Meichen Yan, Yingwen Zhao, Jinglin Li, Luyu Wang, Zhiyuan Yao

**Affiliations:** 1 College of Life Science, Shenyang Normal University, Shenyang 110034, China Shenyang Normal University Shenyang China; 2 Key Laboratory of Eco-environments in Three Gorges Reservoir Region (Ministry of Education), School of Life Sciences, Southwest University, Chongqing 400715, China Southwest University Chongqing China

**Keywords:** Biodiversity, cellar spider, invertebrate, morphology, taxonomy

## Abstract

The spider genus *Pholcus* Walckenaer, 1805 exhibits high diversity in eastern Sichuan, China, and 19 species have been recorded in this area so far. In this study, two additional new species of *Pholcus* from north of the Sichuan Basin are described, *Pholcus jiuzhai* Yan, Li & Yao, **sp. nov.** (♂♀) from the *crypticolens* group and *Pholcus pingwu* Yan, Li & Yao, **sp. nov.** (♂♀) from the *yichengicus* group. In addition, an updated list of all *Pholcus* species from eastern Sichuan is provided.

## ﻿Introduction

*Pholcus* Walckenaer, 1805 is the most diverse genus of the family Pholcidae C.L. Koch, 1850, and it is mainly distributed in the Afrotropical, Palaearctic, Indo-Malayan, and Australasian regions (e.g. Huber 2011; Yao and Li 2012, 2025; WSC 2025). This genus includes 21 species groups and 427 species (Huber 2011; Huber et al. 2018; WSC 2025). China exhibits the greatest diversity of *Pholcus* species (Zhang et al. 2025), and 193 species have been recorded, accounting for 45% of the global total for this group (WSC 2025).

Recently, a series of studies of *Pholcus* have been carried out in northern and central China, based on morphological and molecular data. The survey areas include the Changbai Mountains, the mountainous regions between the Changbai and Yanshan-Taihang Mountains in northeastern China (Lu et al. 2021; Yao et al. 2021; Zhao et al. 2023a; Li et al. 2024, 2025a), the Yanshan-Taihang Mountains and the Lüliang Mountains in northern China (Lu et al. 2022a, b; Zhao et al. 2023b), and the Qinling Mountains in central China (Yang et al. 2024a, b).

**Figure 1.**
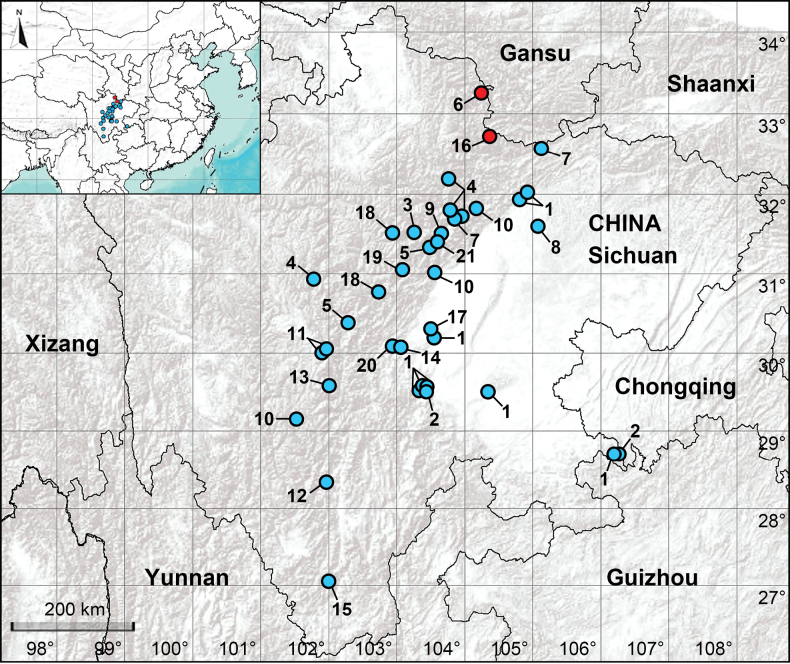
Distribution records of the *Pholcus* spiders from eastern Sichuan. The *bidentatus* group: 1. *P. bidentatus*; 2. *P. kimi*; the *crypticolens* group: 3. *P. aba*; 4. *P. chang*; 5. *P. ganziensis*; 6. *P. jiuzhai* sp. nov.; 7. *P. manueli*; 8. *P. spilis*; 9. *P. wenchuan*; the *yichengicus* group: 10. *P. jiulong*; 11. *P. kangding*; 12. *P. kunming*; 13. *P. luding*; 14. *P. mengding*; 15. *P. miyi*; 16. *P. pingwu* sp. nov.; 17. *P. qingchengensis*; 18. *P. qinghaiensis*; 19. *P. taibaiensis*; 20. *P. yaan*; 21. *P. zhangae*. Blue and red circles represent previously recorded species and new species, respectively.

In 2025, the diversity of *Pholcus* was surveyed in eastern Sichuan, southwestern China, and found 12 species, including five newly described species (Li et al. 2025b). Nevertheless, the majority of these species are distributed in west of the Sichuan Basin. Based on the current distribution pattern of this genus in eastern Sichuan, it is likely that the genus is also present in the highlands of southwest and north of the Sichuan Basin. In this study, two new species of *Pholcus* from north of the Sichuan Basin are described (Fig. 1). An updated list of all *Pholcus* species from eastern Sichuan is also provided (Table 1).

**Table 1.** A list of all *Pholcus* species from eastern Sichuan.

Species	Collection locality	References
***bidentatus* group**		
*P. bidentatus*	Sichuan, Leshan, Emeishan, Huangwan Town, Emeishan Scenic Spot (including two localities)	Huber 2011
	Sichuan, Luzhou, Hejiang County, Zihuai Town	Zhang and Zhu 2009
	Sichuan, Leshan, Emeishan, Huangwan Town, Emeishan Scenic Spot, Fuhu Temple	Zhang and Zhu 2009
	Sichuan, Mianyang, Jiangyou, Zhonghua Town, Laojunshan Scenic Spot	Li et al. 2025b
	Sichuan, Mianyang, Jiangyou, Yongsheng Town, Xinbei Village	Li et al. 2025b
	Sichuan, Chengdu, Pujiang County, Kakadian, Jinma Village	Li et al. 2025b
	Sichuan, Zigong, Cao County, Gaoshiti Forest Park	Li et al. 2025b
*P. kimi*	Sichuan, Luzhou, Hejiang County, Zihuai Town	Zhang et al. 2004
	Sichuan, Leshan, Emeishan, Huangwan Town, Emeishan Scenic Spot, near Fuhu Temple and Baoguo Temple	Zhang et al. 2004
***crypticolens* group**		
*P. aba*	Sichuan, Aba, Li County, Xindianzi	Li et al. 2025b
*P. chang*	Sichuan, Ganzi, Danba County, Geshizha Town, Waba Village	Yao and Li 2012
	Sichuan, Aba, Mao County, Chuanwen Road	Li et al. 2025b
	Sichuan, Aba, Songpan County, G213 Road	Li et al. 2025b
	Sichuan, Aba, Mao County, Mati Village	Li et al. 2025b
*P. ganziensis*	Sichuan, Ganzi, Kangding County, Jintang Town, Jintang River	Yao and Li 2012
	Sichuan, Aba, Wenchuan County, Dayu Grange	Li et al. 2025b
*P. jiuzhai* sp. nov.	Sichuan, Aba, Jiuzhaigou County	This paper
*P. manueli*	Sichuan, Guangyuan, Qingchuan County	Zhang and Zhu 2009
	Sichuan, Aba, Mao County, Chuanwen Road	Li et al. 2025b
*P. spilis*	Sichuan, Mianyang, Zitong County, Wolong Town, Wolongshan Scenic Spot	Zhang and Zhu 2009
*P. wenchuan*	Sichuan, Aba, Wenchuan County, Qingpo Village	Li et al. 2025b
***yichengensis* group**		
*P. jiulong*	Sichuan, Ganzi, Jiulong County, Tanggu Town, Helagou	Tong and Li 2010
	Sichuan, Mianyang, Beichuan County, Dunqing Road, Zaoerping	Li et al. 2025b
	Sichuan, Dujiangyan, Houzhi Grange	Li et al. 2025b
*P. kangding*	Sichuan, Ganzi, Kangding County	Zhang and Zhu 2009
	Sichuan, Ganzi, Kangding County, Paoma Hill	Tong and Li 2010
*P. kunming*	Sichuan, Liangshan, Mianning County, Lizhuang Town, Dishuiyan	Li et al. 2025b
*P. luding*	Sichuan, Ganzi, Luding County, Moxi Town, Hailuogou National Natural Reserve, Caohaizi	Tong and Li 2010
*P. mengding*	Sichuan, Yaan, Yucheng District, Mengdingshan Scenic Spot	Li et al. 2025b
*P. miyi*	Sichuan, Panzhihua, Miyi County, Puwei Town, Pengjiayakou Village	Li et al. 2025b
*P. pingwu* sp. nov.	Sichuan, Mianyang, Pingwu County, Baima Town	This paper
*P. qingchengensis*	Sichuan, Chengdu, Dujiangyan, Qingcheng Mountain	Gao et al. 2002
*P. qinghaiensis*	Sichuan, Yaan, Baoxing County, Yaoji Town, Zegen Village	Tong and Li 2010
	Sichuan, Aba, Li County, Shiziping	Li et al. 2025b
*P. taibaiensis*	Sichuan, Aba, Wenchuan County, Wolong Town, Wolong Natural Reserve	Zhang and Zhu 2009
*P. yaan*	Sichuan, Yaan, Lushan County, Longdongpo	Li et al. 2025b
*P. zhangae*	Sichuan, Aba, Wenchuan County	Zhang and Zhu 2009

## ﻿Materials and methods

Specimens were examined and measured with a Leica M205 C stereomicroscope. Left male palps were photographed. Epigynes were photographed before dissection. Vulvae were photographed after treating them in a 10% warm solution of potassium hydroxide (KOH) to dissolve soft tissues. Images were captured with a Canon EOS 750D wide zoom digital camera (24.2 megapixels) mounted on the stereomicroscope mentioned above and assembled using Helicon Focus v. 3.10.3 image-stacking software (Khmelik et al. 2005). All measurements are given in millimetres (mm). Leg measurements are shown as: total length (femur, patella, tibia, metatarsus, tarsus). Leg segments were measured on their dorsal side. The distribution map was generated with ArcGIS v. 10.2 (ESRI Inc.). The specimens studied are deposited in the College of Life Science, Shenyang Normal University (SYNU) in Liaoning, China.

Terminology and taxonomic descriptions follow Huber (2011) and Yao et al. (2015, 2021). The following abbreviations are used: **a** = appendix, **aa** = anterior arch, **ALE** = anterior lateral eye, **AME** = anterior median eye, **b** = bulb, **da** = distal apophysis, **dp** = distal process, **ds** = dorsal spines, **dsa** = dorso-subdistal apophysis, **e** = embolus, **fa** = frontal apophysis, **kn** = knob, **L/d** = length/diameter ratio, **mb** = median branch, **pa** = proximo-lateral apophysis, **PME** = posterior median eye, **pp** = pore plate, **pr** = procursus, **pra** = proximal apophysis, **psa** = prolatero-subdistal apophysis, **pvp** = prolatero-ventral protrusion, **rdp** = retrolatero-distal process, **rpa** = retrolatero-proximal apophysis, **sl** = subdistal lamella, **u** = uncus, **va** = ventral apophysis, **vp** = ventral protrusion.

## ﻿Taxonomic accounts

### ﻿Family Pholcidae C.L. Koch, 1850

**Subfamily Pholcinae C.L. Koch, 1850**

#### ﻿Genus Pholcus

Taxon classificationAnimaliaAraneaePholcidae

Walckenaer, 1805

F24FA281-4069-516A-955E-5597A74CCF13

##### Type species.

*Aranea phalangioides* Fuesslin, 1775.

### ﻿*Pholcus crypticolens* species group

This species group can be distinguished by the combination of the following characters: male chelicerae with proximo-lateral, distal and frontal apophyses (pa, da, fa); procursus (pr) with dorsal spines (ds); appendix (a) triangular to T-shaped; epigyne sclerotized, with knob (kn) (Huber 2011). The new species below is assigned to this group because its morphological characters are consistent with those as listed above. This species group includes 13 previously described species and is mainly distributed in East Asia (e.g. Huber 2011; Yao and Li 2012; Dong et al. 2016; Li et al. 2025b). Among them, 12 species have been recorded from China. One species is newly described below.

#### ﻿ Pholcus jiuzhai

Taxon classificationAnimaliaAraneaePholcidae

Yan, Li & Yao sp. nov.

94F9EFD7-E762-56FE-8283-AC53C30FA4A6

https://zoobank.org/AA399CDD-1F44-4D99-A45B-8121D5D4E754

Figs 2, 3, 6A

##### Type material.

***Holotype***: China • ♂; Sichuan, Aba, Jiuzhaigou County; 33.2458°N, 104.2516°E; alt. 1456 m; 24 May 2013; X. Jiang leg.; SYNU-Ar00502. ***Paratypes***: China • 7♂; same data as for the holotype; SYNU-Ar00503–509 • 4♀; same data as for the holotype; SYNU-Ar00510–513.

##### Etymology.

The specific name refers to the type locality; it is used as a noun in apposition.

##### Diagnosis.

**Figure 2.**
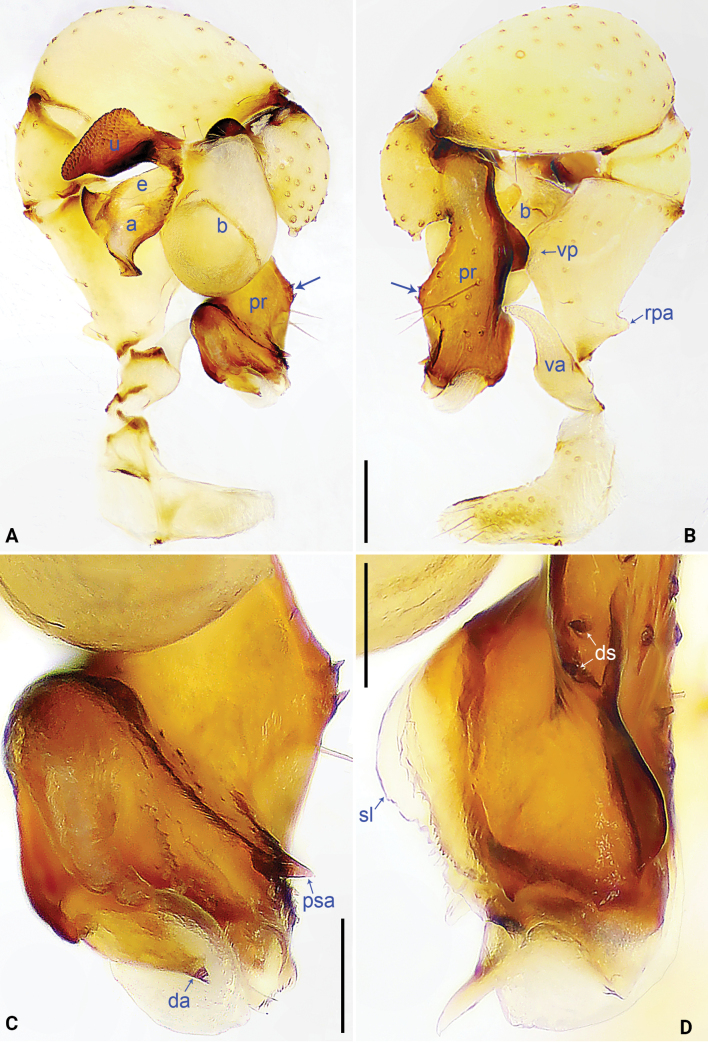
*Pholcus jiuzhai* sp. nov., holotype male. A. Palp, prolateral view; B. Palp, retrolateral view, arrow points to protruding dorso-median part; C. Distal part of procursus, prolateral view; D. Distal part of procursus, dorsal view. Scale bars: 0.20 mm (A, B); 0.10 mm (C, D).

**Figure 3.**
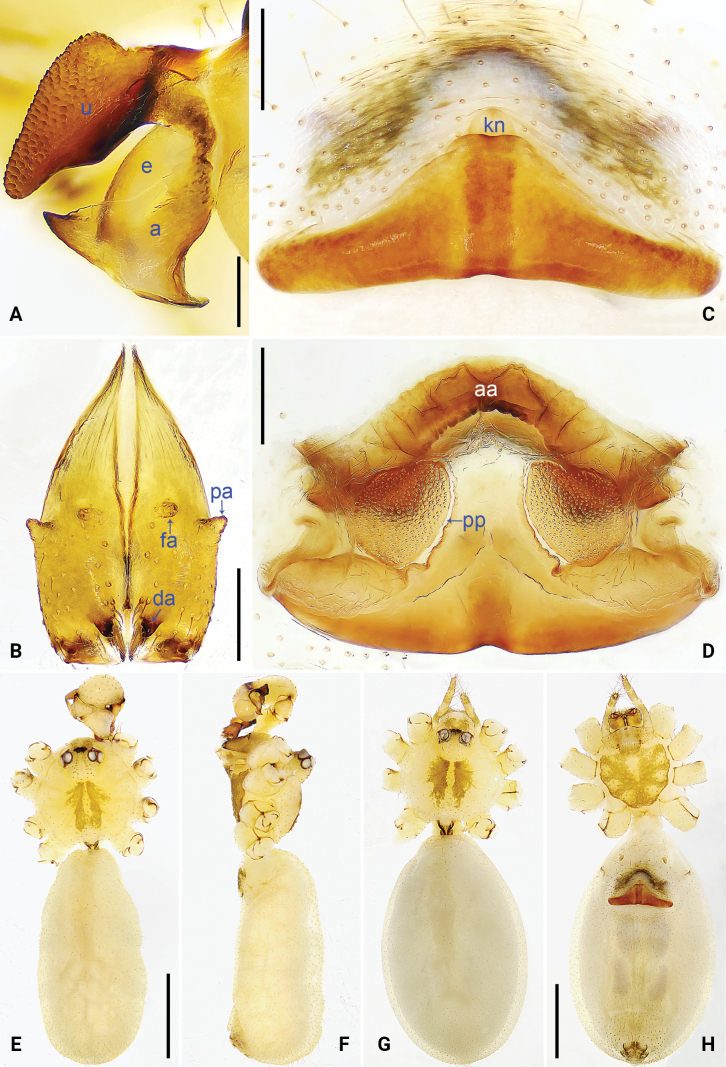
*Pholcus jiuzhai* sp. nov., holotype male (A, B, E, F) and paratype female (C, D, G, H). A. Bulbal apophyses, prolateral view; B. Chelicerae, frontal view; C. Epigyne, ventral view; D. Vulva, dorsal view; E, G. Habitus, dorsal view; F. Habitus, lateral view; H. Habitus, ventral view. Scale bars: 0.10 mm (A); 0.20 mm (B–D); 1.00 mm (E–H).

The new species resembles *P. zichyi* Kulczyński, 1901 (under “*P. crypticolens*” in Zhang and Zhu 2009: 23, fig. 8; Huber 2011: 358, figs 1725, 1726) by having similar male chelicerae (Fig. 3B) and epigyne (Fig. 3C), but can be distinguished by procursus (pr) with weakly sclerotized pointed distal apophysis (da in Fig. 2C vs absent) and dorso-median part of procursus (pr) strongly protruding (arrow in Fig. 2A, B vs very slightly protruding), by proximal part of appendix 1/2 wider than distal part (a in Fig. 3A vs 1/4), by vulval anterior arch medially strongly curved (aa in Fig. 3D vs very slightly curved), and by carapace without marginal brown bands (Fig. 3E, G vs present).

##### Description.

**Male (*holotype*) *measurements***: total length 3.77 (3.86 with clypeus), carapace 1.18 long, 1.29 wide, opisthosoma 2.59 long, 1.28 wide. ***Leg I***: 27.64 (7.02, 0.52, 7.31, 10.83, 1.96), ***leg II***: 18.12 (5.00, 0.52, 4.75, 6.75, 1.10), ***leg III***: 12.80 (3.80, 0.45, 3.06, 4.68, 0.81), ***leg IV***: 16.83 (5.16, 0.42, 4.29, 5.96, 1.00); ***tibia I L/d***: 60. ***Eye interdistances and diameters***: PME–PME 0.20, PME 0.12, PME–ALE 0.04, AME–AME 0.03, AME 0.06. ***Sternum width/length***: 0.88/0.78.

***Colour***: carapace yellowish, with median brown marks; ocular area yellowish; clypeus and sternum yellowish, with brown marks (Fig. 3E, F). Legs yellowish, but brown on patellae and whitish on distal parts of femora and tibiae, without darker rings on femora and tibiae. Opisthosoma yellowish, without spots.

***Body***: as in Fig. 3E, F; ocular area elevated, without eye-stalks.

***Chelicerae***: as in Fig. 3B, with pair of proximo-lateral apophyses (pa), pair of distal apophyses (da) with two teeth each, and pair of frontal apophyses (fa).

***Palp***: as in Fig. 2A, B; trochanter with long (3× longer than wide) ventral apophysis (va); femur with retrolatero-proximal apophysis (rpa) and distinct ventral protrusion (vp). Procursus (pr) simple proximally, but complex distally, with raised prolatero-subdistal edge bearing subdistal lamella (sl) and weakly sclerotized pointed distal apophysis (da), pointed prolatero-subdistal apophysis (psa), and two strong dorsal spines (ds). Bulb: uncus (u) with proximal apophysis (pra, visible in Fig. 6A) and scales; appendix (a) nearly T-shaped; embolus (e) weakly sclerotized, with transparent distal projections (Fig. 6A).

***Legs***: retrolateral trichobothrium on tibia I situated at 3% proximally; legs with short vertical setae on tibiae, metatarsi, and tarsi; tarsus I with 38 distinct pseudosegments.

**Female** (***paratype***, SYNU-Ar00510): similar to male, habitus as in Fig. 3G, H. Total length 4.48 (4.63 with clypeus), carapace 1.32 long, 1.40 wide, opisthosoma 3.16 long, 1.99 wide; ***tibia I***: 6.15; tibia I L/d: 44. ***Eye interdistances and diameters***: PME–PME 0.17, PME 0.13, PME–ALE 0.04, AME–AME 0.05, AME 0.06. ***Sternum width/length***: 0.95/0.88. ***Epigyne*** (Fig. 3C) nearly triangular, with knob (kn). ***Vulva*** (Fig. 3D) with sclerotized, medially strongly curved anterior arch (aa) and pair of nearly round pore plates (pp).

##### Variation.

Tibia I in seven male paratypes (SYNU-Ar00503–509): 6.22–8.72. Tibia I in the other three female paratypes (SYNU-Ar00511–513): 5.71, 6.09, 6.99.

##### Habitat.

Underside of overhang on rocky cliffs in mountainous area.

##### Distribution.

Known only from the type locality (Fig. 1).

### ﻿*Pholcus yichengicus* species group

This species group can be distinguished by the combination of the following characters: male chelicerae with proximo-lateral, distal and frontal apophyses (pa, da, fa); male palpal trochanter apophyses retrolatero-proximally strongly bulged; male palpal tibia with prolatero-ventral protrusion (pvp); procursus (pr) with dorsal spines (ds); appendix (a) usually branched; epigyne sclerotized, with knob (kn) (Huber 2011; Zhu et al. 2018; Yang et al. 2024a). The new species below is assigned to this group because its morphological characters are consistent with those as listed above. This species group includes 51 previously described species and is mainly distributed in central and southern China, as well as northern Thailand (e.g. Huber 2011; Zhu et al. 2018; Lu et al. 2022b; Li et al. 2024; Yang et al. 2024a, b; Li et al. 2025b). Among them, 47 species have been recorded from China. One species is newly described below.

#### ﻿ Pholcus pingwu

Taxon classificationAnimaliaAraneaePholcidae

Yan, Li & Yao sp. nov.

E8BBBBE7-13B0-5889-B9B0-2B911B27DD45

https://zoobank.org/4FF41E4D-7029-46EC-BD84-28D57A62CB05

Figs 4, 5, 6B

##### Type material.

***Holotype***: China • ♂; Sichuan, Mianyang, Pingwu County, Baima Town; 32.7130°N, 104.3770°E; alt. 1801 m; 14 Oct. 2018; Z. Zhang, L. Wang & Z. Fan et al. leg.; SYNU-Ar00514. ***Paratypes***: China • 5 ♂; same data as for the holotype; SYNU-Ar00515–519 • 4 ♀; same data as for the holotype; SYNU-Ar00520–523.

##### Etymology.

The specific name refers to the type locality; it is used as a noun in apposition.

##### Diagnosis.

**Figure 4.**
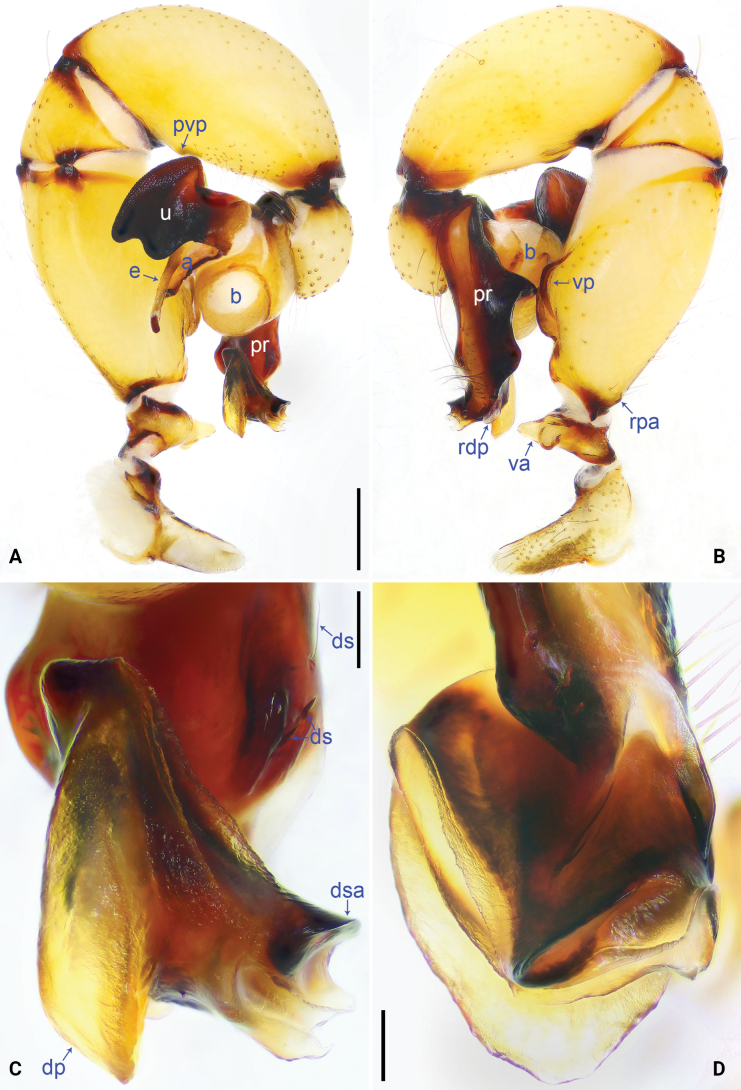
*Pholcus pingwu* sp. nov., holotype male. A. Palp, prolateral view; B. Palp, retrolateral view; C. Distal part of procursus, prolateral view; D. Distal part of procursus, dorsal view. Scale bars: 0.50 mm (A, B); 0.10 mm (C, D).

**Figure 5.**
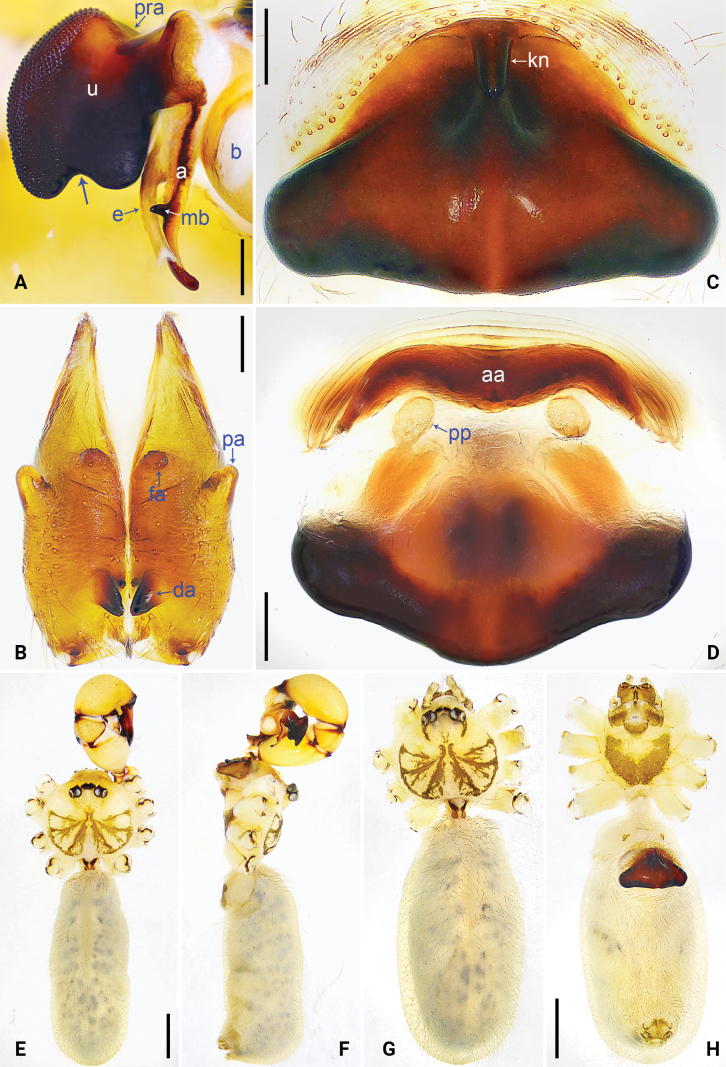
*Pholcus pingwu* sp. nov., holotype male (A, B, E, F) and paratype female (C, D, G, H). A. Bulbal apophyses, prolateral view, arrow points at latero-medially contracted part; B. Chelicerae, frontal view; C. Epigyne, ventral view; D. Vulva, dorsal view; E, G. Habitus, dorsal view; F. Habitus, lateral view; H. Habitus, ventral view. Scale bars: 0.20 mm (A–D); 1.00 mm (E–H).

The new species resembles *P. qingchengensis* Gao, Gao & Zhu, 2002 (only ♂; Zhang and Zhu 2009: 72, fig. 40; Yao and Li 2012: 30, fig. 146) by having similar appendix (a in Fig. 5A) and sclerotized dorso-subdistal apophysis (dsa in Fig. 4C) on procursus (pr), but can be distinguished by procursus (pr) without sclerotized retrolatero-distal apophysis (Fig. 4B vs present) and distal membranous process (dp) of procursus without pointed branch (Fig. 4C vs present), by uncus (u) latero-medially strongly contracted (arrow in Fig. 5A vs slightly contracted), and by male chelicerae with one pair of frontal apophyses (fa in Fig. 5B vs two pairs).

##### Description.

**Male** (***holotype***) ***measurements***: total length 6.82 (6.98 with clypeus), carapace 1.97 long, 2.30 wide, opisthosoma 4.85 long, 1.91 wide. ***Leg I***: 62.60 (15.58, 0.91, 15.83, 26.92, 3.36), ***leg II***: 39.06 (10.71, 0.90, 9.97, 15.51, 1.97), ***leg III***: 26.02 (7.66, 0.83, 6.41, 9.90, 1.22), ***leg IV***: 34.91 (10.38, 0.86, 8.64, 13.30, 1.73); ***tibia I L/d***: 83. ***Eye interdistances and diameters***: PME–PME 0.31, PME 0.20, PME–ALE 0.06, AME–AME 0.09, AME 0.13. ***Sternum width/length***: 1.52/1.32.

***Colour***: carapace yellowish, with brown radiating marks and marginal brown bands; ocular area yellowish; clypeus and sternum yellowish, with brown marks (Fig. 5E, F). Legs yellowish, but dark brown on patellae and whitish on distal parts of femora and tibiae, with darker rings on subproximal, submedian, and subdistal parts of femora and proximal, subproximal, submedian, and subdistal parts of tibiae. Opisthosoma yellowish, with dorsal and lateral spots (Fig. 5E, F).

***Body***: as in Fig. 5E, F; ocular area elevated, each eye triad on top of laterally directed eye-stalks.

***Chelicerae***: as in Fig. 5B, with pair of proximo-lateral apophyses (pa), pair of distal apophyses (da) with two teeth each, and pair of frontal apophyses (fa).

**Figure 6.**
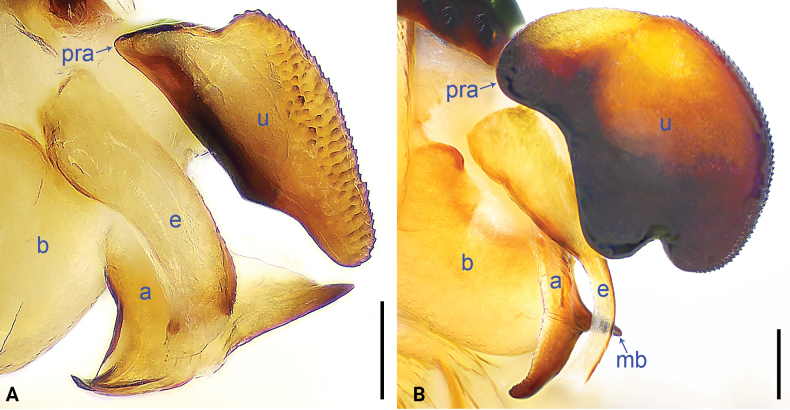
Bulbal apophyses, retrolatero-ventral views. A. *Pholcus jiuzhai* sp. nov.; B. *P. pingwu* sp. nov. Scale bars: 0.10 mm (A); 0.20 mm (B).

***Palp***: as in Fig. 4A, B; trochanter with short (2× longer than wide), retrolatero-proximally strongly bulged ventral apophysis (va); femur with retrolatero-proximal apophysis (rpa) and distinct ventral protrusion (vp); tibia with prolatero-ventral protrusion (pvp). Procursus (pr) simple proximally, but complex distally, with raised prolatero-subdistal edge bearing distal membranous process (dp) and sclerotized dorso-subdistal apophysis (dsa), retrolatero-distal membranous process (rdp), and two strong and one slender dorsal spines (ds). Bulb: uncus (u) latero-medially contracted, with proximal apophysis (pra, visible in Fig. 6B) and distally scaly edge; appendix (a) curved, with distal scales and angular median branch (mb); embolus (e) weakly sclerotized, with transparent distal projections (Figs 5A, 6B).

***Legs***: retrolateral trichobothrium on tibia I situated at 3% proximally; legs with short vertical setae on tibiae, metatarsi, and tarsi; tarsus I with 39 distinct pseudosegments.

**Female** (***paratype***, SYNU-Ar00520): similar to male, habitus as in Fig. 5G, H. Total length 5.70 (6.00 with clypeus), carapace 1.50 long, 1.83 wide, opisthosoma 4.20 long, 2.03 wide; ***tibia I***: 10.83; tibia I L/d: 68. ***Eye interdistances and diameters***: PME–PME 0.21, PME 0.17, PME–ALE 0.07, AME–AME 0.08, AME 0.10. ***Sternum width/length***: 1.19/0.85. Ocular area with lateral brown bands. ***Epigyne*** (Fig. 5C) nearly triangular, strongly sclerotized, with knob (kn). ***Vulva*** (Fig. 5D) with laterally strongly curved anterior arch (aa) and pair of elliptic pore plates (pp).

##### Variation.

Tibia I in five male paratypes (SYNU-Ar00515–519): 12.37–15.96. Tibia I in the other three female paratypes (SYNU-Ar00521–523): 9.62, 10.06, 10.19.

##### Habitat.

Underside of overhang on rocky cliffs in mountainous area.

##### Distribution.

Known only from the type locality (Fig. 1).

## ﻿Discussion

Our study has identified two new species of *Pholcus* from north of the Sichuan Basin. To date, 21 species have been documented in eastern Sichuan. Recent research has revealed that Old World *Pholcus* species are believed to have originated in northern Indochina and southeastern Central Asia (the eastern Neo-Tethyan region), and the greatest species diversity is concentrated in southeast of the Xizang Plateau (Yao and Li 2025). Notably, eastern Sichuan falls within this high-diversity region. However, the diversity of *Pholcus* in eastern Sichuan is currently mainly concentrated in west and north of the Sichuan Basin. Current knowledge of the distribution of *Pholcus* in eastern Sichuan suggests that this genus is highly likely to also be present in the highlands of southwestern Sichuan Basin. Therefore, additional targeted and concerted collecting efforts should be directed towards that part of the Sichuan Basin to further explore its potential biodiversity.

## Supplementary Material

XML Treatment for Pholcus

XML Treatment for Pholcus jiuzhai

XML Treatment for Pholcus pingwu

## ﻿Additional information

### Use of AI

No use of AI was reported.

### Author ORCIDs

Meichen Yan https://orcid.org/0009-0001-7306-3323

Yingwen Zhao https://orcid.org/0009-0002-5991-1354

Jinglin Li https://orcid.org/0009-0008-3932-309X

Luyu Wang https://orcid.org/0000-0002-5250-3473

Zhiyuan Yao https://orcid.org/0000-0002-1631-0949

### Data availability

All of the data that support the findings of this study are available in the main text.
